# Supplementary material for: Association of PM2.5 and Its Chemical Compositions with Metabolic Syndrome: A Nationwide Study in Middle-Aged and Older Chinese Adults
Source: Int J Environ Res Public Health. 2022 Nov 8;19(22):14671. doi: 10.3390/ijerph192214671 (PMC9690751; doi:10.3390/ijerph192214671)
Supplement: Supplementary file 1 [file ijerph-19-14671-s001.zip › ijerph-2001574-supplementary.pdf]

Table S1. The definition of the MetS from different organization.

Index	Population	China	NCEP/ATP III	AHA	CDS	IDF
WC	Men	>85cm	>90cm	>102cm	>90cm	>94cm
	Women	>80cm	>80cm	>88cm	>85cm	>80cm
BP	All	SBP ≥130 mmHg or DBP ≥85 mmHg	SBP ≥130 mmHg or DBP ≥85 mmHg or on antihypertensive medication	SBP ≥130 mmHg or DBP ≥85 mmHg or on antihypertensive medication	SBP ≥130 mmHg or DBP ≥85 mmHg or diagnosed hypertension	SBP ≥130 mmHg or DBP ≥85 mmHg or taking anti-hypertensive medication
FBG	All	>100 mg/dL	≥150 mg/dL or antidiabetic medication	≥100 mg/dL or antidiabetic medication	FBG ≥100 mg/dL or 2h-glucose ≥140 mg/dL or diagnosed diabetes mellitus	≥100 mg/dL or taking antidiabetic medication
FTG	All	>150 mg/dL	≥150 mg/dL or on medication for elevated TG	≥150 mg/dL or on medication for elevated TG	≥150 mg/dL	≥150 mg/dL or taking medication for reduced TG
HDL-C	Men	<40 mg/dL	<40 mg/dL or taking medication for reduced HDL-C	<40 mg/dL or taking medication for reduced HDL-C	<40 mg/dL	<40 mg/dL or taking medication for reduced HDL-C
	Women	<50 mg/dL	<50 mg/dL or taking medication for reduced HDL-C	<50 mg/dL or taking medication for reduced HDL-C	<40 mg/dL	<50 mg/dL or taking medication for reduced HDL-C

Abbreviations: NCEP/ATP III, the US National Cholesterol Education Programme Adult Treatment Panel III guidelines; AHA, American Heart Association; CDS, Chinese Diabetes Society; IDF, the International Diabetes Federation; SBP, systolic blood pressure; DBP, diastolic blood pressure; FBG, fasting blood glucose; HDL-C, high density lipoprotein cholesterol; MetS, metabolic syndrome; FTG, fasting triglyceride; WC, waist circumference. MetS was defined as if a participant presented with three or more of the abovementioned risk factors.

Table S2. The average concentration of the $PM_{2.5}$ and its chemical constituent in different exposure window.

Air pollutants	Mean	SD	Min	P_5	P_{25}	Median	P_{75}	P_{95}	Max	Lag
$PM_{2.5}$	52.12	21.62	18.38	22.30	34.30	48.44	65.92	90.59	97.66	2y
BC	2.51	0.77	0.93	1.30	1.90	2.35	3.10	3.83	4.20	2y
NH_4^+	7.86	3.41	2.61	2.93	4.90	7.55	10.37	13.64	14.37	2y
NO_3^-	11.29	5.58	3.07	3.37	6.44	10.49	15.51	20.82	21.95	2y
OM	12.61	4.65	4.70	5.73	8.94	12.00	15.80	21.60	22.80	2y
SO_4^{2-}	9.75	3.69	2.91	4.65	6.78	9.42	12.63	16.15	17.11	2y
$PM_{2.5}$	54.16	22.69	17.89	22.53	36.05	51.24	70.29	94.29	101.29	3y
BC	2.63	0.82	0.92	1.33	1.98	2.48	3.31	3.93	4.39	3y
NH_4^+	8.15	3.59	2.52	2.94	4.96	7.89	10.89	14.14	14.85	3y
NO_3^-	11.66	5.86	3.01	3.36	6.53	11.01	16.21	21.74	22.70	3y
OM	13.03	4.86	4.56	5.68	9.17	12.45	16.81	21.91	23.54	3y
SO_4^{2-}	10.24	3.95	2.85	4.61	7.07	10.17	13.66	16.92	17.75	3y
$PM_{2.5}$	54.93	23.02	18.18	22.69	36.53	51.29	71.12	95.56	102.88	4y
BC	2.69	0.84	0.94	1.33	2.03	2.53	3.44	4.09	4.46	4y
NH_4^+	8.29	3.66	2.53	2.93	5.15	8.12	11.10	14.46	15.03	4y
NO_3^-	11.78	5.93	3.08	3.32	6.61	11.16	16.32	22.06	22.87	4y
OM	13.18	4.90	4.61	5.75	9.28	12.43	16.94	21.83	23.63	4y
SO_4^{2-}	10.48	4.07	2.89	4.65	7.15	10.41	13.97	17.54	18.30	4y
$PM_{2.5}$	55.66	23.10	18.20	23.60	36.89	52.97	72.79	96.71	103.30	5y
BC	3.65	1.59	0.94	1.70	2.42	3.30	4.38	6.94	7.53	5y
NH_4^+	8.32	3.65	2.49	2.91	5.18	8.15	11.26	14.35	14.98	5y
NO_3^-	11.86	5.93	3.06	3.31	6.70	10.99	16.53	22.04	22.79	5y
OM	13.50	5.06	4.57	5.78	9.43	12.85	17.50	22.53	23.78	5y
SO_4^{2-}	10.70	4.17	2.87	4.66	7.24	10.59	14.34	17.78	18.71	5y

Abbreviations: BC: Black Carbon; NH_4^+: Ammonium; NO_3^-: Nitrate; OM: organic matter; SO_4^{2-}: Sulfate.

Table S3. The summary of the statistics of the concentration of $PM_{2.5}$ constitution in different region.

East [1](μg/m^3)									
Constitution	Mean	SD	Min	P_5	P_{25}	Median	P_{75}	P_{95}	Max
$PM_{2.5}$	57.24	22.58	24.52	25.32	33.78	59.12	79.46	86.23	89.85
BC	2.65	0.80	1.45	1.53	1.91	2.53	3.47	3.80	3.99
NH_4^+	8.40	3.42	3.49	3.72	4.72	9.17	11.46	13.03	13.39
NO_3^{2-}	12.46	5.60	4.41	4.72	5.99	13.98	17.42	19.88	20.40
OM	13.53	4.98	6.62	7.09	8.87	12.75	18.11	21.39	21.70
SO_4^{2-}	10.52	3.57	5.08	5.30	6.88	11.11	13.98	15.44	15.95
Middle [2](μg/m^3)									
$PM_{2.5}$	56.40	16.43	26.93	31.05	43.68	54.35	65.67	86.38	93.27
BC	2.51	0.59	1.59	1.60	2.06	2.31	2.89	3.54	3.83
NH_4^+	8.93	2.41	4.07	4.86	6.80	8.56	10.74	12.68	14.03
NO_3^{2-}	13.25	4.11	5.17	6.57	9.82	12.67	16.06	19.80	21.38
OM	13.31	3.39	7.55	7.95	10.88	12.59	15.61	20.12	20.52
SO_4^{2-}	10.62	2.71	5.56	6.27	8.60	10.69	12.30	15.19	16.61
West [3](μg/m^3)									
$PM_{2.5}$	36.52	12.77	19.84	20.41	23.53	35.41	44.76	61.46	62.06
BC	2.03	0.54	0.98	1.24	1.60	1.93	2.38	2.96	3.05
NH_4^+	5.49	2.37	2.76	2.85	3.34	5.11	7.50	9.84	10.65
NO_3^{2-}	7.14	3.40	3.07	3.30	4.08	6.77	9.53	13.41	14.38
OM	9.54	3.06	4.95	5.28	6.86	9.17	11.81	15.01	15.41
SO_4^{2-}	7.21	2.59	3.07	3.87	5.02	6.43	9.27	12.40	12.62
Northeast [4](μg/m^3)									
$PM_{2.5}$	42.72	8.22	26.85	26.85	38.89	45.52	49.64	50.55	50.55
BC	1.97	0.39	1.26	1.26	1.69	2.04	2.31	2.42	2.42
NH_4^+	5.93	1.53	2.88	2.88	4.20	6.45	7.09	7.43	7.43
NO_3^{2-}	8.97	2.50	3.30	3.30	6.59	9.76	10.67	11.38	11.38
OM	10.00	2.29	6.22	6.22	8.14	10.18	12.21	12.55	12.55
SO_4^{2-}	7.11	1.50	4.86	4.86	5.08	7.60	8.61	8.62	8.62

Notes:

[1]East including Beijing, Tianjin, Hebei, Shanghai, Jiangsu, Zhejiang, Fujian, Shandong, Guangdong, and Hainan.

[2] Middle including Shanxi, Anhui, Jiangxi, Henan, Hubei, and Hunan.

[3] West including Inner Mongolia, Guangxi, Chongqing, Sichuan, Guizhou, Yunnan, Xizang, Shaanxi, Gansu, Qinghai, Ningxia, and Xinjiang.

[4] Northeast including Liaoning, Jilin, and Heilongjiang.

Abbreviations: BC: Black Carbon; NH_4^+: Ammonium; NO_3^-: Nitrate; SO_4^{2-}: Sulfate. OM: organic matter.

Table S4. The association of metabolic syndrome (MetS) with an IQR incremental change in 1-year average $PM_{2.5}$ and its constitution in different model.

PM and its composition	Different Model	Exposure window	Odds Ratio	P value
$PM_{2.5}$	Crude Model	1y	1.23(1.16,1.3)	<0.001
$PM_{2.5}$	Model II	1y	1.25(1.16,1.34)	<0.001
$PM_{2.5}$	Model III	1y	1.27(1.17,1.37)	<0.001
$PM_{2.5}$	Model IV	1y	1.27(1.17,1.37)	<0.001
$PM_{2.5}$	Model V	1y	1.27(1.17,1.37)	<0.001
BC	Crude Model	1y	1.2(1.14,1.27)	<0.001
BC	Model II	1y	1.23(1.15,1.32)	<0.001
BC	Model III	1y	1.25(1.16,1.36)	<0.001
BC	Model IV	1y	1.25(1.15,1.36)	<0.001
BC	Model V	1y	1.25(1.16,1.36)	<0.001
NH_4^+	Crude Model	1y	1.15(1.09,1.22)	<0.001
NH_4^+	Model II	1y	1.18(1.1,1.27)	<0.001
NH_4^+	Model III	1y	1.21(1.12,1.31)	<0.001
NH_4^+	Model IV	1y	1.21(1.12,1.31)	<0.001
NH_4^+	Model V	1y	1.21(1.12,1.31)	<0.001
NO_3^-	Crude Model	1y	1.19(1.13,1.26)	<0.001
NO_3^-	Model II	1y	1.22(1.13,1.31)	<0.001
NO_3^-	Model III	1y	1.25(1.15,1.35)	<0.001
NO_3^-	Model IV	1y	1.24(1.14,1.35)	<0.001
NO_3^-	Model V	1y	1.25(1.15,1.35)	<0.001
OM	Crude Model	1y	1.2(1.14,1.27)	<0.001
OM	Model II	1y	1.22(1.14,1.31)	<0.001
OM	Model III	1y	1.25(1.15,1.35)	<0.001
OM	Model IV	1y	1.25(1.15,1.35)	<0.001
OM	Model V	1y	1.25(1.15,1.35)	<0.001
SO_4^{2-}	Crude Model	1y	1.17(1.1,1.24)	<0.001
SO_4^{2-}	Model II	1y	1.19(1.11,1.29)	<0.001
SO_4^{2-}	Model III	1y	1.23(1.13,1.33)	<0.001
SO_4^{2-}	Model IV	1y	1.22(1.12,1.33)	<0.001
SO_4^{2-}	Model V	1y	1.22(1.12,1.33)	<0.001

Notes: Crude Model: No adjustment. Model II: Crude Model+ age, sex, urbanicity, educational level, marriage status, physical activity. Model III: Model II+ cooking fuel type, heating fuel type. Model IV: Model III+ smoke. Mode V (Fully Model): Model IV+ drinking. Abbreviations: BC: Black Carbon; NH_4^+: Ammonium; NO_3^-: Nitrate; OM: organic matter; SO_4^{2-}: Sulfate.

Table S5. The association of metabolic syndrome (MetS) with an IQR incremental change in $PM_{2.5}$ and its constitution in different exposure window.

PM and its composition	Exposure Window	Odds Ratio	P value
$PM_{2.5}$	1y	1.27(1.17,1.37)	<0.001
$PM_{2.5}$	2y	1.25(1.16,1.35)	<0.001
$PM_{2.5}$	3y	1.26(1.16,1.36)	<0.001
$PM_{2.5}$	4y	1.25(1.16,1.35)	<0.001
$PM_{2.5}$	5y	1.16(1.04,1.30)	<0.001
BC	1y	1.25(1.16,1.36)	<0.001
BC	2y	1.25(1.15,1.36)	<0.001
BC	3y	1.24(1.14,1.35)	<0.001
BC	4y	1.24(1.14,1.36)	<0.001
BC	5y	1.21(1.14,1.29)	<0.001
NH_4^+	1y	1.21(1.12,1.31)	<0.001
NH_4^+	2y	1.22(1.12,1.32)	<0.001
NH_4^+	3y	1.22(1.12,1.33)	<0.001
NH_4^+	4y	1.21(1.11,1.32)	<0.001
NH_4^+	5y	1.21(1.11,1.32)	<0.001
NO_3^-	1y	1.25(1.15,1.35)	<0.001
NO_3^-	2y	1.25(1.15,1.36)	<0.001
NO_3^-	3y	1.25(1.15,1.36)	<0.001
NO_3^-	4y	1.24(1.14,1.35)	<0.001
NO_3^-	5y	1.24(1.14,1.35)	<0.001
OM	1y	1.25(1.15,1.35)	<0.001
OM	2y	1.24(1.15,1.34)	<0.001
OM	3y	1.25(1.15,1.36)	<0.001
OM	4y	1.25(1.15,1.36)	<0.001
OM	5y	1.24(1.14,1.35)	<0.001
SO_4^{2-}	1y	1.22(1.12,1.33)	<0.001
SO_4^{2-}	2y	1.21(1.12,1.32)	<0.001
SO_4^{2-}	3y	1.22(1.12,1.33)	<0.001
SO_4^{2-}	4y	1.22(1.11,1.33)	<0.001
SO_4^{2-}	5y	1.22(1.11,1.33)	<0.001

Notes: The model was adjusted for age, sex, urbanicity, educational level, marriage status, smoke, drinking, cooking fuel type, heating fuel type, and physical activity. Abbreviations: BC: Black Carbon; NH_4^+: Ammonium; NO_3^-: Nitrate; OM: organic matter; SO_4^{2-}: Sulfate. Lag1 refers to the average exposure concentration of the previous year from the date of investigation.

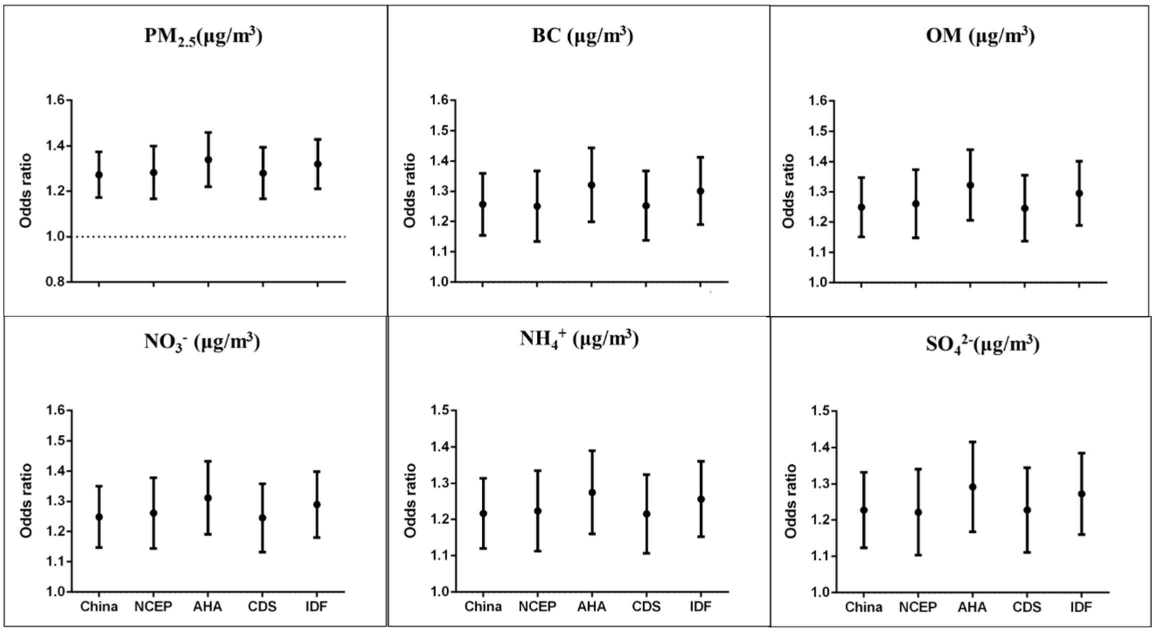

Figure S1. The association of metabolic syndrome (MetS) with an IQR incremental change in 1-year average $PM_{2.5}$ and its constitution by different definition. The bars show main effect estimates and 95% confidence intervals. The model was adjusted for age, sex, urbanicity, educational level, marriage status, smoke, drinking, cooking fuel type, heating fuel type, and physical activity. Abbreviations: BC: Black Carbon; NH_4^+: Ammonium; NO_3^-: Nitrate; OM: organic matter; SO_4^{2-}: Sulfate. NCEP, the US National Cholesterol Education Programme; AHA, American Heart Association; CDS, Chinese Diabetes Society; IDF, the International Diabetes Federation.
